# Using a cascade approach to assess condom uptake in female sex workers in India: a review of the Avahan data

**DOI:** 10.1186/s12889-018-5842-6

**Published:** 2018-07-20

**Authors:** Renay Weiner, Micah Fineberg, Bridget Dube, Prabuddhagopal Goswami, Shajan Mathew, Gina Dallabetta, Saul Johnson

**Affiliations:** 1 School of Public Health, University of the Witwatersrand, Johannesburg, South Africa; 2 Genesis Analytics, Johannesburg, South Africa; 3 FHI 360, New Delhi, India; 4 Bill and Melinda Gates Foundation, Washington, DC USA

**Keywords:** Female sex workers, India, Avahan, HIV prevention cascades, Condom

## Abstract

**Background:**

The Avahan India AIDS Initiative was implemented to provide HIV prevention services to key populations including female sex workers (FSWs) who carry the burden of India’s concentrated HIV epidemic. Established in 2003 and handed over to the Indian government in 2009, the Initiative included peer-led outreach education, condom promotion and distribution and STI treatment. This study aimed to determine if HIV prevention cascades could be generated using routine monitoring and evaluation data from the Avahan program and to assess their value in identifying and responding to program gaps for FSWs.

**Methods:**

Two data sources were used namely the Integrated Behavioural and Biological Assessment reports and the Centralized Management Information System dataset. Indicators selected for the cascades were: FSWs at risk, belief that HIV can be prevented, condom access and consistent condom use with an occasional partner. Six districts were selected and stratified by HIV prevalence at baseline and two cascades were generated per district reflecting changes over time.

**Results:**

Consistent condom use with occasional partners in this population increased in all six districts during program implementation, with statistically significant increases in four of the six. No patterns in the cascades were detected according to HIV prevalence either at baseline (2005) or at follow-up (2009). Cascades were able to identify key programmatic bottlenecks at baseline that could assist with focusing program efforts and direct resources at district levels. In some districts the belief that HIV could not be prevented contributed to inconsistent condom use, while in others, low levels of condom access were a more important barrier to consistent condom use.

**Conclusion:**

This HIV prevention cascade analysis among FSWs in India suggests that cascades could assist in identifying program gaps, focus intervention efforts and monitor their effect. However, cascades cannot replace a detailed understanding of the multiple factors at individual, community and structural levels that lead to consistent condom use in this key population. Careful indicator selection coupled with innovative data collection methods will be required. Pilot projects are proposed to formally evaluate the value of HIV prevention cascades at district level.

## Background

In 2006, an estimated 1.7 million people aged 15–49 years were living with HIV in India, representing the country with the third largest number of HIV positive people worldwide [1]. India’s HIV burden is concentrated in key populations such as female sex workers (FSWs) [2]. The HIV prevalence of FSWs in India is estimated to be 2.2% and varies greatly between states [3]. It is well documented that the HIV epidemic in India is driven by a high HIV prevalence among FSWs and their clients [4, 5]. Although the commercial exchange of sex between consenting heterosexual adults is not illegal in India, police extort and arrest FSWs, invoking the Immoral Trafficking Prevention Act or, more frequently, ‘public nuisance’ laws [6].

In response to this, the Avahan India AIDS Initiative was established by the Bill &Melinda Gates Foundation in 2003 [7]. The program was implemented in six Indian states: Andhra Pradesh (one state during Avahan, but has since divided into Andhra Pradesh and Telengana), Tamil Nadu, Karnataka, Maharashtra, Manipur and Nagaland, comprising 29 districts. These states accounted for approximately 83% of HIV cases in India at the time [7]. HIV prevention services were provided to about 300,000 people from high risk groups, including peer-led outreach education, condom promotion and distribution, and clinical services to treat sexually transmitted infections (STIs), as well as structural and community mobilisation interventions [7]. After a staged and managed transition in 2009, the program was handed over to the Indian Government [8].

Avahan was supported by major monitoring and evaluation elements. Extensive biological and behaviour data was collected in target groups through sample surveys conducted at least twice during the project period. Routine monthly monitoring data reported on size estimations, operational and infrastructure elements, service provision, service uptake and community activities [9]. Data was fed back into the program to inform resource allocation and intervention planning [10].

As the focus on HIV prevention intensifies, the question has arisen whether the cascade approach can be applied to HIV prevention programs for their monitoring and advocacy. Typically, cascades are based on HIV treatment monitoring data, which focus on getting people living with HIV to a point of viral suppression. HIV prevention cascades focus on the steps required to prevent HIV infection and successfully implement HIV prevention programs. Prevention cascades include demand-side interventions that focus on increasing awareness, acceptability and uptake of prevention interventions, supply-side interventions that make prevention interventions more accessible and available, and adherence interventions that support ongoing adoption and compliance with prevention behaviours or products. The prevention cascade provides estimates of the proportions of targeted population lost at each implementation step. This allows identification of gaps for further planning and focus to ensure successful program implementation.

Since the approach for HIV prevention cascades is in its infancy, various groups have sought to focus on different prevention cascades. Some prevention cascades seek to integrate social, behavioural and biomedical interventions to quantify the number of individuals lost at each intervention step [11]. This is used to support program design and to facilitate resource allocation [12]. Client-centric cascades explore how those at risk can avoid infection by perceiving their risk and adopting the necessary intervention. Intervention-centric cascades make interventions available to priority populations and then observe how the intervention is taken-up [13]. These cascades are hard to construct with existing data since much of the data on perception of risk is not widely available. Simpler cascades, based on more widely available monitoring data and similar to treatment cascades are also in use [14].

In the past, prevention cascades have been successful in exploring the performance of interventions to prevent mother-to-child transmission of HIV [15–18]. Other examples include tracking the impact of HIV testing and counselling on condom use, voluntary medical male circumcision uptake, and reducing the number of partners in rural Zimbabwe [13]. There is global interest in investigating the use of cascades for other prevention tools among key population programs [11, 19]. Prevention cascades are more complicated than treatment cascades because prevention involves multiple interventions, often simultaneous, and individuals move in and out of periods of risk.

Addressing HIV among key populations is critical for curbing the HIV epidemic. HIV prevalence is highest in key populations, and yet access to services is relatively low [20]. Policy and structural barriers, stigma and discrimination make it difficult for key populations to access services; there is a need to provide dedicated and tailored services for this group [14]. Non-health facility based data systems are thus necessary to understand if key populations are being reached and taking up services in an effective way. Through the use of cascades, data can generate meaningful insights for currently uninfected individuals with highly varied behaviours and needs [21]. This does not detract from the importance of monitoring the use of condoms amongst those who are HIV positive to prevent onward transmission, even though this is not captured by the cascades. Applying HIV prevention cascades to large scale prevention programs for key populations, such as Avahan, could determine where the program exerts effects and identify constraints to efficacy.

The objective of this study was to determine whether meaningful HIV prevention cascades, focused on condom uptake among female sex workers, could be generated using both routine monitoring data and evaluation data from the Avahan program. This will assist future programs in designing data collection systems which lend themselves to producing prevention cascades. Ultimately, regular use of these cascades will allow programs to identify inefficiencies for resolution, resulting in more effective HIV prevention program implementation.

## Methods

### Inclusion criteria

**Fig. 1:**
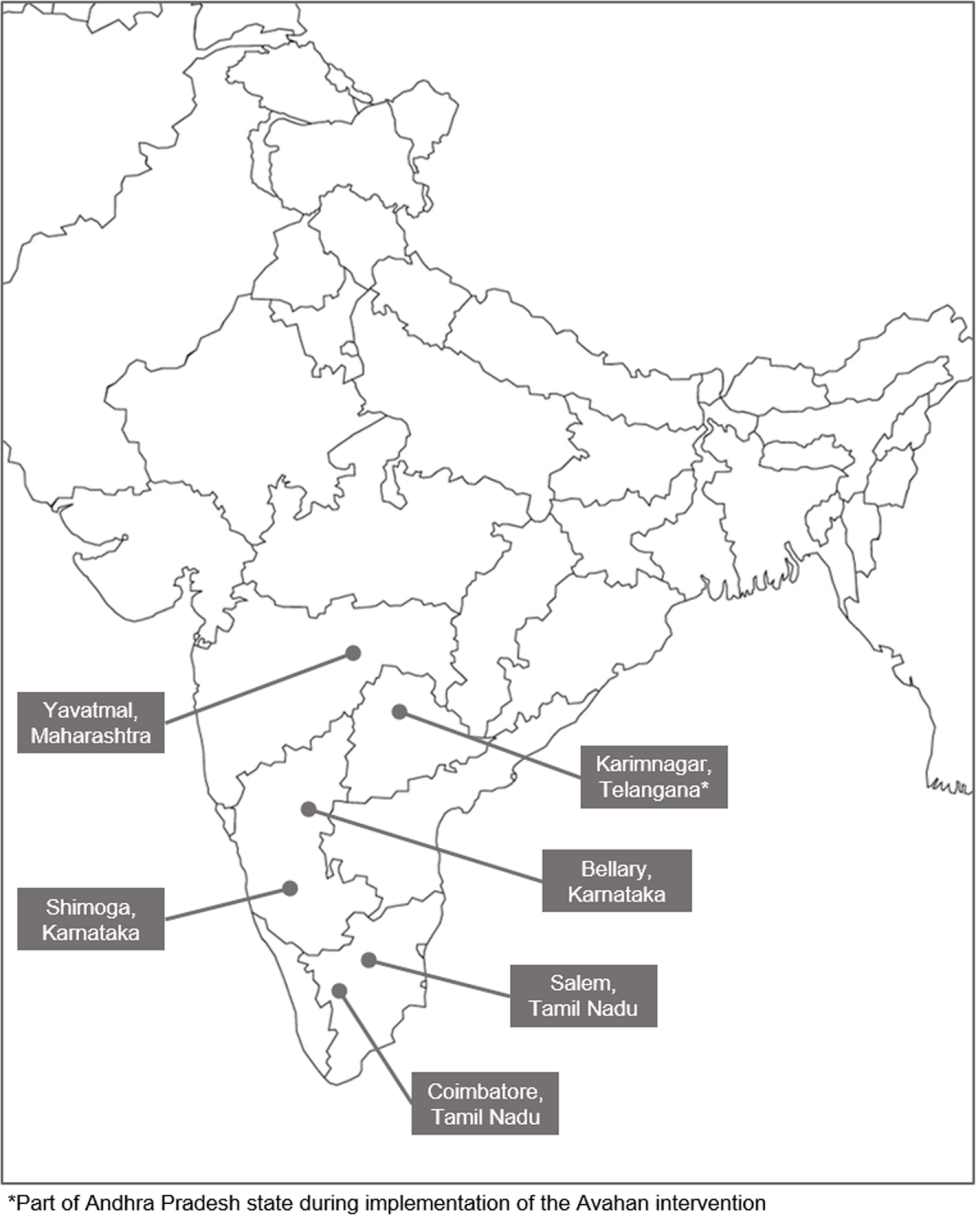
Map with districts included in the analysis. Districts were included if they experienced either a significant decline or no change in HIV prevalence, if the Avahan Initiate was the sole implementer of prevention programs, and if Integrated Behavioural and Biological Assessment data was available for both rounds of the survey. Source: Adapted from Bruce Jones Design Inc. [31]

Districts selected for the cascade analysis included those that matched the following criteria: 1) experienced either a decrease or no change in HIV prevalence, 2) Avahan was the sole implementer of FSW programs so that the role of other programs could be excluded, and 3) IBBA data were available. Based on these criteria, six districts (Yevatmal, Karimnagar, Bellary, Salem, Shimoga, Coimbatore) were included into the final analysis (Fig. 1).

In this study, FSWs (18+) were either brothel-based (working/living/operating in brothels in red light/brothel areas) or non-brothel-based (soliciting male clients on the street or in other non-brothel settings), who sold sex in exchange for cash at least once in the last one month. The Avahan program comprised a package of interventions, but we focused on developing cascades for condom distribution and promotion through peer-led educators and NGOs given both the importance of condom use in this population and the available data.

### Data analysis

The first step of our analysis was to assess if there was sufficient and appropriate data for constructing a condom use prevention cascade. Data was reviewed from existing evaluation reports and routine data spreadsheets. Two datasets were available for review, the Integrated Behavioural and Biological Assessments (IBBA) dataset and the Centralized Management Information System (CMIS) dataset [22]. IBBAs were conducted in 2005–2007 and 2009–2010 in all six Avahan states in 29 districts [23]. These population-based surveys collected data on socio-demographic characteristics, HIV risk behaviours and condom use. Follow-up biological testing measured HIV and STI prevalence. A total of 22,915 FSWs were covered by the intervention. For the districts selected in our cascades, the sample sizes were small with less than 450 FSWs per district [24]. Programmatic monitoring data was captured by NGOs and peer-educators on a monthly basis, and used for standardized reporting. The CMIS reported on a set of 39 indicators, including service provision, service uptake, community activities, and operational and infrastructure elements [25]. Combined, these two datasets contained the indicators necessary to develop a condom use prevention cascade. The IBBA data reported on ideational factors, condom access, condom use, and HIV prevalence for districts. Indicators lacking in the IBBA data were successfully recovered in the CMIS dataset, such as the number of FSWs estimated per district and condom distribution [22].

These data were modelled for a pilot district, Karimnagar. Two methods were applied to the pilot; 1) a client-centric cascade, which included condom access data from IBBA surveys and 2) a system-centric cascade that included condom supply data from CMIS. For both methods, two cascades were constructed, at the beginning (2005) and the end of the program (2009). An accurate system-centric cascade could not be generated for condom supply due to inconsistencies in the CMIS data at the start of the program. Thus, a client-centric cascade was developed, using ‘consistent condom use with occasional clients’ as the key condom use indicator. This was defined as use of a condom at every sex act with an occasional client. We considered this a suitable indicator for condom use for HIV prevention, as condoms are only effective when used consistently, and this behaviour is difficult to adopt.

**Fig. 2:**
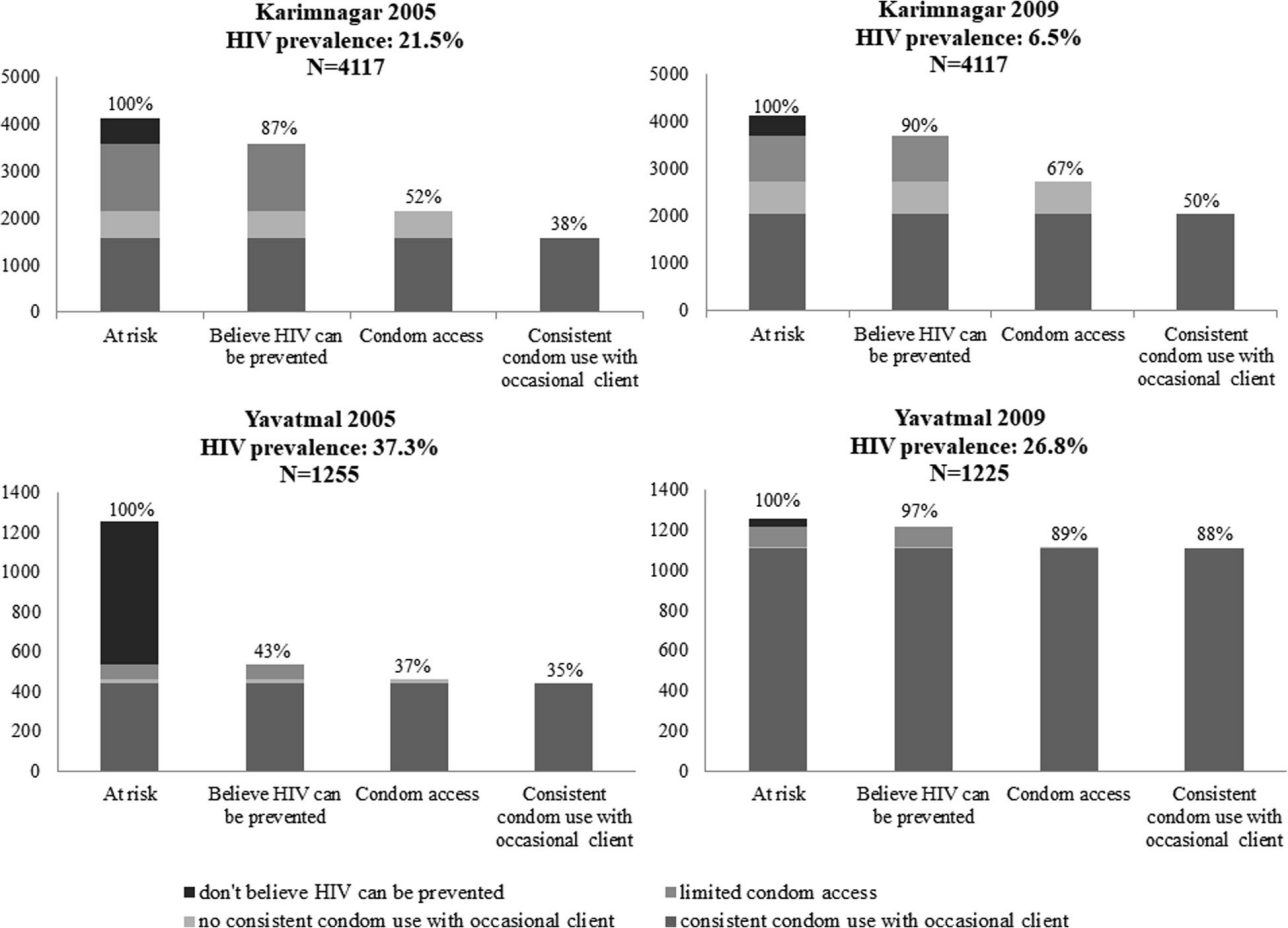
HIV prevention cascades for high baseline HIV prevalence districts: 2005 and 2009*.* Patterns of consistent condom use with occasional clients at baseline (2005) and after implementation of Avahan (2009) for districts with a high baseline HIV prevalence (> 20%), namely Karimnagar (baseline HIV prevalence of 21.5%; *N* = 4117) and Yavatmal (baseline HIV prevalence of 37.3%; *N* = 1255)

**Fig. 3:**
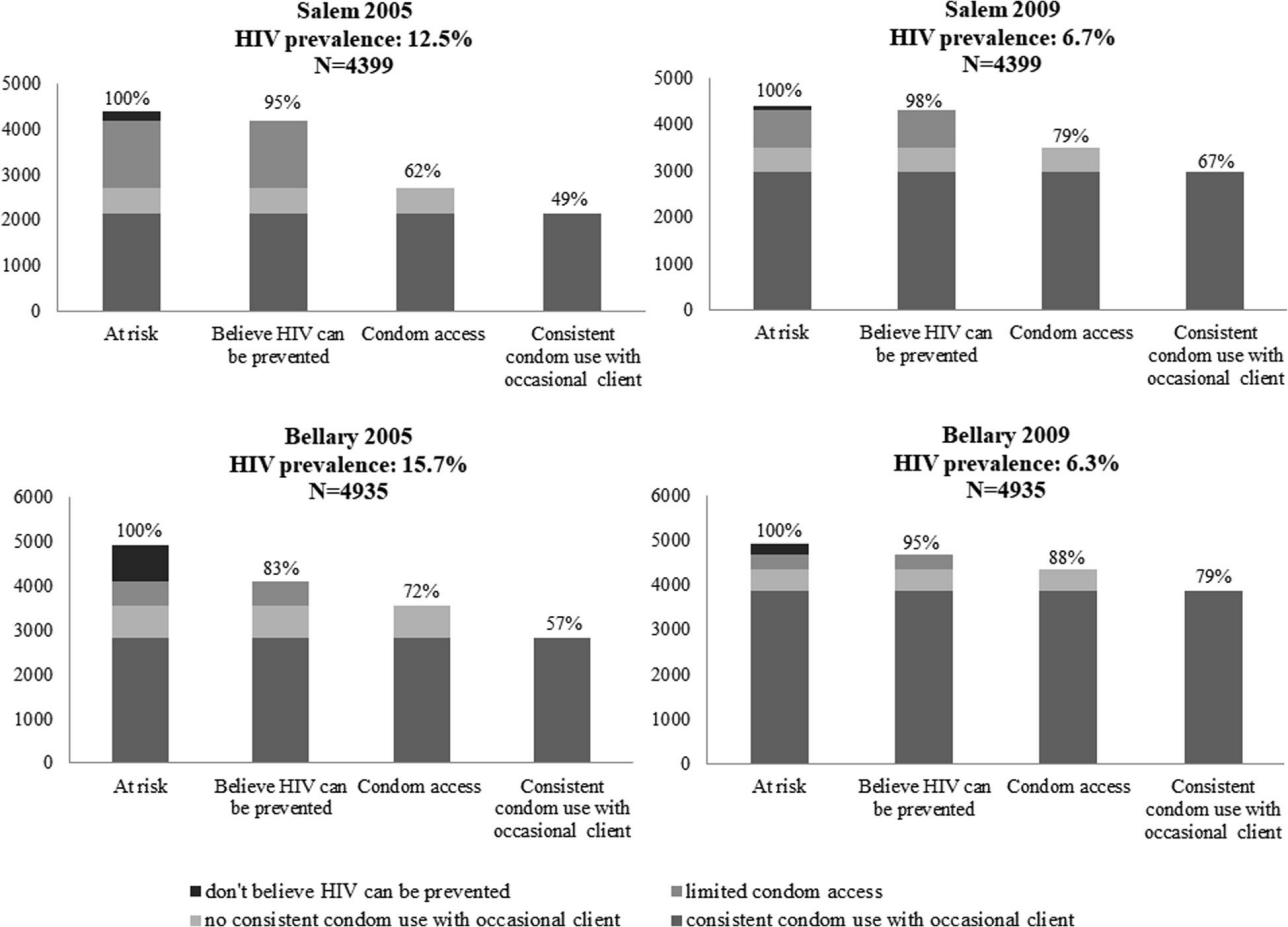
HIV prevention cascades for medium baseline HIV prevalence districts: 2005 and 2009. Patterns of consistent condom use with occasional clients use at baseline (2005) and after implementation of Avahan (2009) for districts with a medium baseline HIV prevalence (10–20%), namely Salem (baseline HIV prevalence of 12.5%; *N* = 4399) and Bellary (baseline HIV prevalence of 15.7%; *N* = 4935)

**Fig. 4:**
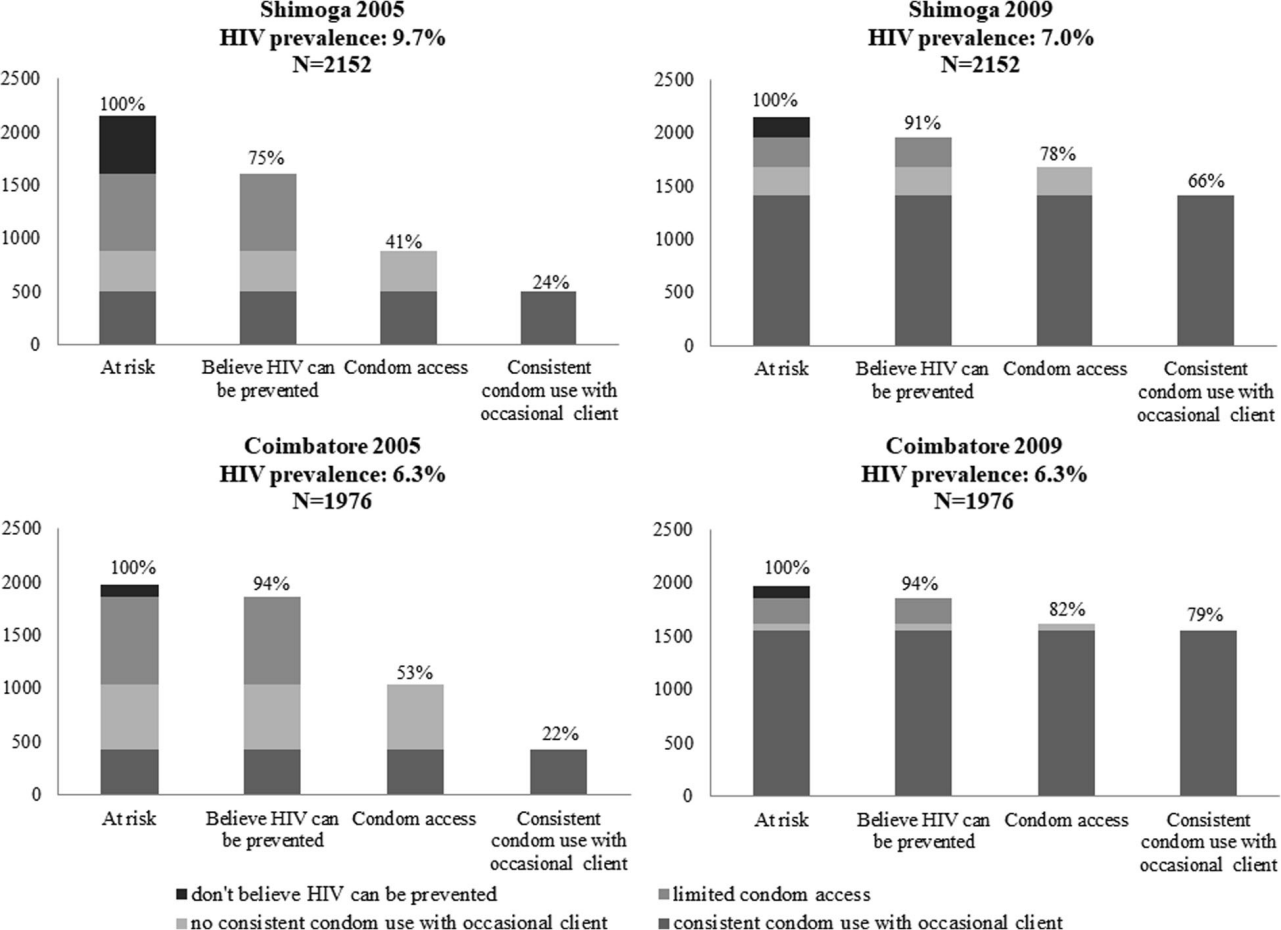
HIV prevention cascades for low baseline HIV prevalence districts: 2005 and 2009. Patterns of consistent condom use with occasional clients use at baseline (2005) and after implementation of Avahan (2009) for districts with a low baseline HIV prevalence (< 10%), namely Shimoga (baseline HIV prevalence of 9.7%; *N* = 2152) and Coimbatore (baseline HIV prevalence of 6.3%; *N* = 1976)

The client-centric cascade sought to measure demand side variables, namely risk perception and belief in prevention, and the supply side variable of condom access and adherence as measured by self-reported consistent condom use with an occasional client. These were based on existing survey questions, rather than questions specifically designed for a prevention cascade. Risk perception was considered if the FSWs felt at risk of infection with HIV. This indicator was however excluded from the final analysis, since in several districts risk perception decreased over time. While there are possible reasons for this, it was difficult to fit this with the cascade models. Knowledge was determined by the belief that there are things a person can do to prevent getting infected with HIV/AIDS. Respondents were taken to have access to condoms if they had been given condoms by a peer-educator from an NGO even though condoms are also supplied free from government and socially marketed by PSI. Uptake of condoms was measured in terms of reported consistent condom use with occasional clients by FSW. The total number of FSWs captured in the CMIS dataset was taken as the highest number in the follow-up period and used for the `At risk’ indicator. IBBA data, namely `believe HIV can be prevented’, `condom access’ and `consistent condom use with occasional client’ were available as percentages, which were applied to the `At risk’ number to generate numbers to construct the cascades. These data were entered into Microsoft Office (2016) Excel to generate cascade graphs for all six districts (Figs. 2, 3, and 4).

## Results

### HIV prevalence and condom use rates

HIV prevalence and consistent condom use rates amongst FSWs over the period of implementation in the six districts are presented in Table 1.

**Table 1:** HIV prevalence amongst FSWs in six districts in 2005 and 2009

District	State	HIV prevalence % (CI) 2005	HIV prevalence % (CI) 2009	Consistent condom use with occasional partner % (CI) 2005	Consistent condom use with occasional partner % (CI) 2009
Yevatmal	Maharashtra	37.3 (25.1–51.2)	26.8 (19.1–36.1)	95 (90.5–97.1)	99 (96.6–99.6)
Karimnagar	Andhra Pradesh	21.1 (14.1–30.4)^a^	6.5 (4.2–10.1)^a^	73 (65.9–79.3)	75 (68.8–80.4)
Bellary	Karnataka	15.7 (11.7–20.6)	6.3 (10.9–18.1)	79 (73.7–83.2)^a^	89 (84.9–92.3)^a^
Salem	Tamil Nadu	12.5 (8.4–18.2)	6.7 (3.9–11.3)	79 (72.1–84.2)^a^	95 (90.6–97.1)^a^
Shimoga	Karnataka	9.7 (6.8–13.6)	9.0 (6.2–12.8)	57 (50.2–63.0)^a^	84 (78.4–88.1)^a^
Coimbatore	Tamil Nadu	6.3 (3.5–11.0)	6.3 (3.6–10.8)	41 (35.2–46.9)^a^	96 (93.1–97.6)^a^

^a^Statistically significant change

It is evident that in all six districts, HIV prevalence rates were either stable or decreased between 2005 and 2009. Those districts with relatively low HIV prevalence rates at baseline, Shimoga and Coimbatore, showed the least decrease in prevalence. Conversely, there were greater decreases in high prevalence districts such as Yevatmal and Karimnagar. While the IBBA reports do not present *p*-values, non-overlapping confidence intervals suggest a statistically significant decrease in HIV prevalence in Karimnagar district [3].

High consistent condom use with occasional partners at baseline correlates positively with HIV prevalence rates indicating higher condom uptake in the districts with high HIV prevalence. Of note is that consistent condom use with occasional partners in this population increased in all districts over time, and in some cases substantially. Statistically significant increases, as determined by non-overlapping confidence intervals, were achieved in four of the districts (highlighted in Table 1).

### HIV prevention cascades

The HIV prevention cascades for six districts are presented for 2005 and 2009 and are stratified according to baseline HIV prevalence rates of FSWs into high (> 20%), medium (10–20%) and low (< 10%) (Figs. 2, 3, and 4) [3].

#### Baseline HIV prevalence > 20% in Karimnagar and Yavatmal

Figure 2 shows cascades for Karimnagar and Yavatmal districts and indicates two different patterns both at baseline and after implementation of the Avahan intervention. In Yavatmal district, a relatively high proportion of FSW did not believe HIV could be prevented, and this shifted substantially after four years in parallel with an increase in consistent condom use with occasional clients. Limited access to condoms appears to have contributed minimally to low consistent condom use in this district. In contrast, lack of access to condoms appears to have been important in Karimnagar at baseline, which was partially overcome in 2009.

#### Baseline HIV prevalence 10–20% in Salem and Bellary

HIV prevalence rates were similar in these two districts and halved during the study period (Fig. 3). In Salem, limited condom access appears to have been a substantial barrier to consistent use, which improved over the implementation phase. However, in Bellary, belief that HIV could not be prevented, combined with limited condom access, contributed to inconsistent condom use which also improved over time.

#### Baseline HIV prevalence < 10% in Shimoga and Coimbatore

In both of these districts, consistent condom use with an occasional client increased markedly over time. In Shimoga, overcoming limited condom access and lack of belief in HIV being preventable were both important factors at baseline, that decreased after the intervention. For Coimbatore, the cascades suggest that condom access was the main factor influencing consistent use with occasional clients, and removing this barrier led to improved condom use (Fig. 4).

## Discussion

HIV prevention cascades for FSWs in six districts in India were generated with existing Avahan program data to assess their value in identifying and responding to program gaps. The cascades were produced at two points in time, 2005 and 2009, in each district and were stratified by the FSW HIV prevalence rates in 2005.While cascades have previously been developed for FSWs in Uganda and in Kenya, the Avahan district cascades are the first, to our knowledge, that attempt to show shifts in programs indicators over time [13]. Consistent condom use with an occasional partner was selected as a key measure of HIV prevention programs amongst FSWs.

With increasing interest and investment in using cascades to intensify HIV prevention globally, this exercise has helped to highlight where HIV prevention cascades could be useful and where their limitations lie, particularly for FSW programs. No patterns in the cascades were detected according to HIV prevalence either at baseline or at follow-up, suggesting that in this population cascades were not helpful as a monitoring tool for predicting, tracking or evaluating decreases in HIV prevalence. Conversely, the cascades were able to identify key programmatic bottlenecks at baseline that could assist with focusing program efforts and directing resources at district levels. For example, in Yavatmal in 2005, the cascade could have detected a need to strengthen behaviour change programs that increase knowledge and beliefs about HIV prevention, while in Karimnagar, Salem, Shimoga and Coimbatore districts a focus on improving condom access by FSW would have strengthened prevention efforts. With reliable and regular data from routine monitoring and sample surveys, repeated cascade generation could track changes over time and for FSWs, and monitor condom supply and distribution indicators that contribute to consistent condom use.

Discussions at recent cascade workshops have considered how best to construct cascades that will be used for program management [6, 13]. For simplicity and usability, a four bar cascade has been recommended for program purposes and various indicators have been considered in different settings. Client-centric condom cascades for FSWs in Uganda included population at risk, perceived risk, ever used condoms and consistent condom use, while intervention-centric cascade replaced risk perception with condom availability [8]. The LINKAGES project, reporting on lessons learnt from accelerated programs for key populations in four African countries, proposes cascades and visual tools based on readily available data, to support program managers which could be usefully applied to other geographical contexts [14].

The Avahan district cascades presented here combined belief about prevention with condom access, as reported by FSWs. Including perceived risk or belief about prevention ensures that programs do not overlook non-health system factors that influence condom uptake. Another issue under discussion is the use of tailored versus standardized cascades. Districts may derive greater benefit from cascades if indicators are selected by districts and are aligned to their programs. Additional standardized cascades for aggregating data to higher levels such as states/provinces, could be added. A behaviour change intervention is likely to select knowledge, attitudes and belief indicators, whereas for a health systems intervention condom supply and coverage will be important. For example, in Nairobi, intervention-centric cascades were developed to provide condom availability information for managers and/or distribution centres [6]. At a minimum, an ideational/demand indicator, access/supply indicator and effective use indicator would be desirable [13]. While aligning cascades to programs is important, this should not limit interventions but rather offer guidance on how to reach defined outcomes. A more nuanced and multi-level approach to interventions that considers community and structural barriers rather than a single factor is likely to be more effective than targeting individual indicators serially. Schaefer et al., proposed an explanatory framework to guide the interpretation of prevention cascades, by recognising the importance of partners, peer norms and social norms, as well as structural factors, in determining uptake of an intervention [6].

HIV prevention cascades have been challenged given that unlike treatment, prevention needs between and within populations are varied [15]. However, prevention cascades may be more useful for key populations than for general populations, particularly for behavioural outcomes, since key populations arguably have a minimum set of common prevention needs to be met. For FSWs these include access to a readily available supply of condoms, and the knowledge and skills required for consistent uptake.

There are several limitations to using HIV prevention cascades for FSW programs. The factors that lead to condom use are unlikely to be linear as reflected by the bars of the cascade, and unlike treatment cascades, prevention actions are not necessarily consequent upon one another [15]. For example, good knowledge, beliefs and intentions may not necessarily lead to condom use if the client is unwilling and/or financial factors lead the FSW to engage in condom-less sex. Qualitative evidence from Mumbai and Thane indicates that sex workers’ agency to use condoms continues to be compromised by gatekeepers, notably brothel owners. Young women who are new entrants to the sex industry are particularly vulnerable to the control of brothel owners who may prevent their access to outreach programs [26]. The environment within which risk behaviour takes place needs to be considered and addressed for successful HIV prevention amongst FSWs.

Similarly, enabling factors in the environment that go beyond the key intervention need to be considered when using cascades since they cannot take account of, nor measure, the range of relevant interventions and influences that may have an important bearing on condom use. For example, in Kanartaka, FSWs who had high exposure to community mobilisation, defined as being a member of collective or peer group, were more than four times more likely to use a condom at last sex with an occasional client, compared to those who were not part of a collective. This was over and above the contribution of peer education [27].

Another concern relates to measurement of interventions. While the measure of condom access may indicate that a high proportion of FSWs received a condom, the question used, `have you been given condoms from peers or outreach workers from the Avahan NGO?’ does not capture the intensity of the intervention and is not sufficiently designed to detect regular condom access [8]. Survey questionnaires could be revised to take account of the need for more detailed intervention data to increase the validity of condom access measures and in particular to avoid overestimating condom access.

For cascades to be useful, high quality, inexpensive and easily obtainable data is required. Usefulness will hinge on the ability of program managers to interpret the cascades. Sources of regular behavioural data, including risk perception, knowledge and attitudes need to be considered since these are usually collected through sample surveys which are logistically complicated, expensive and take time to generate results. More innovative ways to collect ideational data, such as digital methods, therefore need to be explored. Mobile phones have become prominent in the Indian FSW environment to solicit and maintain clients, as well as connect to social networks and stay safe [28]. Since phones are widely used and can provide real-time data these could enable regular HIV prevention measurement amongst FSWs. However, the acceptability and feasibility would need to be carefully assessed, and confidentiality concerns would need to be addressed. While there is a lack of evidence for the use of mHealth tools to support FSW programs, digital devices have successfully collected behavioural data for other key populations [29].

While measuring consistent condom use is key in tracking FSW program progress, ways to overcome the inherent reporter bias in condom use data needs to be addressed, particularly for women heavily exposed to HIV prevention programs. Bandewar et al. found that the intense focus on condom use may increase social desirability in reporting condom use amongst sex workers in India. Indeed, client reported consistent condom use with occasional FSW clients was lower than that reported by the FSW [26]. Given this limitation, Bradley et al. examined condom availability in Kanartaka by estimating the proportion of FSW sex acts that were potentially protected by condoms and found that condom use was lower than reported by FSWs in 2004,before the Avahan program [30]. Since face-to-face interviews overestimate condom use by 8–20% compared to polling booth methods [30], the relative anonymity of mobile phones may have the added advantage of limiting reporter bias.

## Conclusion

This analysis has shown that HIV prevention cascades, focused on condom uptake in FSWs, can be generated using both routine monitoring data and evaluation data. HIV prevention is a priority for FSW, and strategies to strengthen programs need to be actively appraised and tested. There could be substantial value in introducing cascades as a visualisation tool at the outset of programming to identify bottlenecks, focus intervention efforts and monitor their effect. This analysis has highlighted specific gaps where more focused implementation of the Avahan programme would have been beneficial (e.g. strengthening interventions for demand, supply or adherence in difference districts, respectively). Cascades would not replace a detailed understanding of the key population and the multiple factors at individual, community and structural levels that lead to uptake of the desired intervention. Careful indicator selection, particularly at district level, coupled with innovative data collection methods such as mobile phones, will be required to ensure success. To this end, pilot projects which integrate program managers at the outset, would be helpful to formally design HIV prevention cascades and evaluate their value in strengthening FSW programs at district level.

## Abbreviations

AIDS: Acquired Immuno-Deficiency Syndrome
CMIS: Centralised Management Information System
FSW: Female Sex Worker
HIV: Human Immuno-Deficiency Virus
IBBA: Integrated Behavioural and Biological Assessment
ICMR: Indian Council of Medical Research
NGO: Non-Governmental Organisation
STI: Sexually Transmitted Infection
